# Artificial intelligence in healthcare: applications, challenges, and future directions. A narrative review informed by international, multidisciplinary expertise

**DOI:** 10.3389/fdgth.2025.1644041

**Published:** 2025-11-06

**Authors:** Ata Mohajer-Bastami, Sarah Moin, Suhaib Ahmad, Ahmed R. Ahmed, Sjaak Pouwels, Shahab Hajibandeh, Wah Yang, Chetan Parmar, Mohammad Kermansaravi, Miriam Khalil, Ali Waleed Khalid, Ameer Khamise, David Rawaf, Farzad Hosseini, Anurag Agarwal, Anil Lala, Shafi Ahmed, Bijendra Patel, Barbara Fyntanidou, Richard Egan, Stavroula G. Mougiakakou, Dominik Andreas Jakob, Vincent Ribordy, Wolf E. Hautz, Aristomenis K. Exadaktylos

**Affiliations:** 1 Brompton Primary Care Network, London, United Kingdom; 2 Department of General Surgery, East Surrey Hospital, London, United Kingdom; 3 Department of Surgery, Health Education and Improvement Wales (HEIW), Wales, United Kingdom; 4 Department of Surgery, Imperial College London, London, United Kingdom; 5 Department of Surgery, Bielefeld University—Campus Detmold, Detmold, Germany; 6 Department of Intensive Care Medicine, Elisabeth-Tweesteden Hospital, Tilburg, Netherlands; 7 Department of General Surgery, Morriston Hospital, Swansea, United Kingdom; 8 Department of Surgery, The First Affiliated Hospital of Jinan University, Guangzhou, China; 9 Department of Surgery, Whittington Hospital, London, United Kingdom; 10 Department of Surgery, University College London, London, United Kingdom; 11 Department of Surgery, Apollo Hospitals, Telengana, India; 12 Department of Surgery, Division of Minimally Invasive and Bariatric Surgery, Hazrat-e Fatemeh Hospital, School of Medicine, Iran University of Medical Sciences, Tehran, Iran; 13 St James University Hospital, Leeds, United Kingdom; 14 School of Medicine, University of Buckingham, Buckingham, United Kingdom; 15 WHO Collaborating Centre for Public Health Education and Training, Imperial College London, London, United Kingdom; 16 Kingsmill Hospital, Nottingham, United Kingdom; 17 Department of General Surgery, Betsi Cadwaladr University Health Board, Wales, United Kingdom; 18 Bart’s Health NHS Trust, London, United Kingdom; 19 Director, University Emergency Department, Aristotle University of Thessaloniki, Thessaloniki, Greece; 20 ARTORG Center for Biomedical Engineering Research, AI in Health and Nutrition, University of Bern, Bern, Switzerland; 21 Department of Emergency Medicine, Inselspital University Hospital of Bern, Bern, Switzerland; 22 Department of Emergency Medicine, HFR Fribourg—Cantonal Hospital, Villars-sur-Glâne, Switzerland

**Keywords:** artificial intelligence, machine learning, deep learning, large language models, generative AI, digital health, healthcare, surgery

## Abstract

**Objectives:**

This narrative review evaluates the role of artificial intelligence (AI) in healthcare, summarizing its historical evolution, current applications across medical and surgical specialties, and implications for allied health professions and biomedical research.

**Methods:**

We conducted a structured literature search in Ovid MEDLINE (2018–2025) using terms related to AI, machine learning, deep learning, large language models, generative AI, and healthcare applications. Priority was given to peer-reviewed articles providing novel insights, multidisciplinary perspectives, and coverage of underrepresented domains.

**Key findings:**

AI is increasingly applied to diagnostics, surgical navigation, risk prediction, and personalized medicine. It also holds promise in allied health, drug discovery, genomics, and clinical trial optimization. However, adoption remains limited by challenges including bias, interpretability, legal frameworks, and uneven global access.

**Contributions:**

This review highlights underexplored areas such as generative AI and allied health professions, providing an integrated multidisciplinary perspective.

**Conclusions:**

With careful regulation, clinician-led design, and global equity considerations, AI can augment healthcare delivery and research. Future work must focus on robust validation, responsible implementation, and expanding education in digital medicine.

## 1 Introduction

Artificial Intelligence (AI) has been a rapidly growing scientific field which effectively aims to create machine technology to perform tasks that normally require human intelligence. Artificial intelligence has risen in visibility due to its significant capability in performing tasks requiring normally human cognition, using deep learning models. The use of artificial intelligence has been noticed across different sectors from the creative arts to silence. The complex mergence with healthcare systems proves highly promising but it also comes with its own challenges, with the potential to improve patient outcomes while raising many ethical and regulatory challenges (1).

Healthcare is undergoing a transformational shift due to growing demands, healthcare costs and increasingly strained systems (2). Particularly after the recent Covid-19 pandemic which seemed to worsen existing health inequalities (3), there has never seemed a better opportunistic time to implement an emerging technology to augment clinical practice. From potential time and cost savings in drug discovery and medical diagnostics (4, 5), to revolutionary insights into genomic sequencing and disease susceptibility (5), AI has been recently emerging into all areas of healthcare from preventative medicine and public health to acute hospital medicine and surgery. Since the development of machine learning and deep learning, applications of AI in healthcare have expanded beyond an algorithm-based model of medicine to a more personalised approach (6).

Recently, improved AI systems have led to the potential of AI improving or even replacing current functions of doctors (7). There are, however, several barriers that restrict its universal adoption, including lack of transparency in AI algorithms, which goes against the medical ethos of clinical medicine relying on transparency in decision making with current use of evidence-based medicine in clinical practice.

The motivation for this review stems from the rapid pace of AI adoption in non-medical sectors compared to healthcare, despite healthcare being one of the areas with the greatest need for innovation. While prior reviews have addressed AI in specific domains such as radiology or surgery, few have comprehensively examined its cross-disciplinary impact, including underrepresented fields such as allied health professions and generative AI. This paper aims to provide a multidisciplinary synthesis of AI applications across medicine, surgery, allied health, and biomedical research. Furthermore, we also aimed to critically evaluate the limitations and regulatory challenges of implementation. And finally we aimed to propose future research directions that can guide safe, equitable, and responsible AI integration. The review is structured to first present the history and evolution of AI, followed by its clinical and research applications, limitations, and finally, directions for future integration into healthcare systems.

This paper aims to discuss the history of AI and its increasingly prominent role in clinical practice, particularly in recent history. It discusses the role of AI across various domains of healthcare including medical and surgical specialties, as well as health prevention and health promotion. This paper also addresses the current limitations to the use of AI in clinical practice and explores possible solutions. Furthermore, this review considers potential future applications and strategies for more streamlined implementation into wider healthcare systems.

In addition, this review examines underrepresented domains such as allied healthcare professions and Generative AI (GenAI). In contrast to the traditional focus on physician-led applications, this review explores the role of AI in physiotherapy, speech therapy, nutrition and mental health. We also aim to explore the role of large language models (LLMs) in automating and improving documentation, communication and decision making. This provides a realistic oversight on the possible future integration of AI in healthcare.

We conducted a literature search in Ovidmedline up to July 2025. Search terms included combinations of “artificial intelligence,” “machine learning,” “deep learning,” “large language models,” “generative AI,” “digital health,” and “healthcare” alongside specialty-specific terms such as “surgery,” “radiology,” “mental health,” “allied health professions,” “ethics,” “regulation,” and “clinical applications.” Boolean operators (AND, OR) were used to refine results. Priority was given to peer-reviewed articles published between 2018 and 2025 that provided novel insights, multidisciplinary perspectives, and coverage of underrepresented domains such as allied healthcare and AI regulation.

## 2 Understanding AI and its history

**Figure 1:**
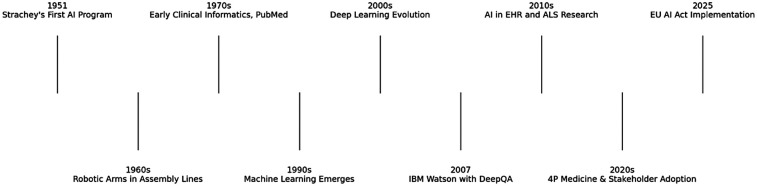
Timeline of major milestones in AI and healthcare.

AI has significantly evolved since the first AI program was developed by Christopher Strachey in 1951. It was initially mainly an academic research topic and in the following two decades, there were great innovative advances in engineering such as electronic arms in assembly lines and the first simple robot able to follow basic instructions (6). Despite this progression, medicine was slow to adopt this technology during this period. There were however major advancements in medicine during this time which would help establish the foundations of AI in medicine in the future; these include the development of medical record systems and clinical informatics databases (8). The web-based search engine PubMed was created in this time by the National Library of Medicine and was a fundamental digital resource which facilitated the acceleration of biomedicine as we know it (8) (Figure 1).

The following decades saw a shift towards Machine Learning, which is a subfield of AI which focuses on pattern identification and analysis, aiming to improve machines with experience from data sets. Subsequently following this, Natural Language Processing, another AI subfield which involves computers extracting data from human language and based on that information making decisions was developed (9).

From the late 1990s and 2000s onwards, Machine Learning had progressed into Deep Learning, a system of multi-layer neural networks which enables machines to learn and make decisions on their own, acting similarly to the human brain (9). The 2000s started to see seminal advancements in AI. In 2007, the International Business Machines Corporation (IBM) developed Watson, a question-answering system that relied on a technology called DeepQA, which used Natural Language Processing to analyse data and generate answers (10). This system was easily maintainable and was more cost-effective.

Utilising information stored in electronic medical records as well as other available electronic resources, DeepQA technology opened the door and revolutionised new possibilities in clinical decision making backed by evidence-based medicine (11). This was exactly demonstrated by Bakkar et al. (12) who used this technology in amyotrophic lateral sclerosis (ALS) to identify RNA-binding proteins that were altered.

With previous limitations in computing power and funding, applications of AI such as this was non-existent before. However, in more recent times with enhancement of computational power, greater volume of data and further funding; there is a more optimistic view for the use of AI in medicine^8^.

From diagnostics to operational management of healthcare, these recent emergences have made stakeholders more invested in its use in clinical medicine and beyond. The general population has generally met this with great enthusiasm as it gives more patient autonomy by enabling the “4P” model of medicine (predictive, preventive, personalised and participatory) (13), in a way that was previously difficult to achieve.

This paper will now consider specific areas of medicine whereby AI has been utilised in an effective manner with better patient outcomes.

## 3 Current applications of artificial intelligence in modern clinical practice

### 3.1 AI in surgical specialities

**Figure 2:**
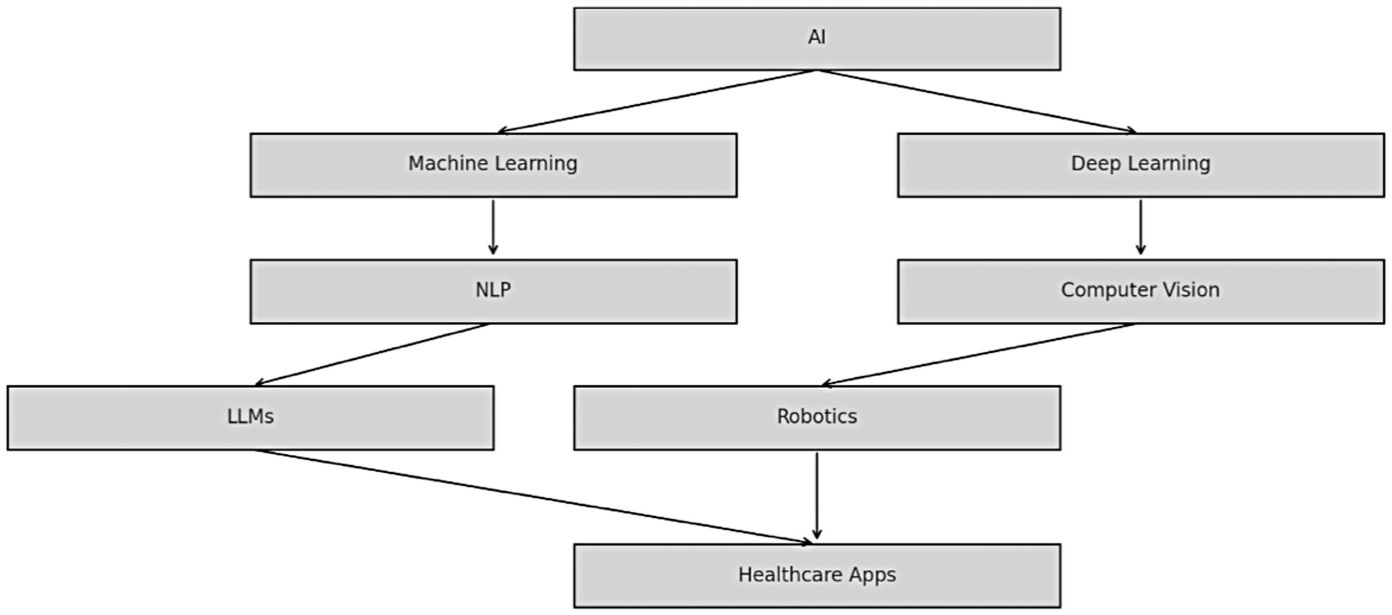
Timeline of major milestones in AI and healthcare.

Surgeons are often needing to make complex decisions under the constraints of time pressure and diagnostic uncertainty which can have a great effect on patient outcomes (Figure 2; Table 1).

**Table 1:** Summary of AI applications in healthcare.

Specialty	Applications	Benefits	Challenges
General Surgery	Intra-operative guidance, risk stratification	Enhanced precision, reduced errors	Training needs, regulatory concerns
Gastrointestinal Endoscopy	Polyp detection, lesion classification	Higher adenoma detection rates	False positives, trust building
Oncology	Predicting treatment response and survival	Personalized treatment, improved outcomes	Data diversity, model transparency
Radiology	Automated image analysis, diagnosis support	Faster diagnostics, reduced workload	Over-reliance, bias, legal concerns
Cardiology	Arrhythmia detection, risk calculators	Early diagnosis, better screening	False positives, device reliability
Neurology	Seizure detection, motor symptom tracking	Continuous monitoring, accurate tracking	Privacy concerns, model reliability
Mental Health	CBT tools, behavioral pattern analysis	Early diagnosis, treatment stratification	Overgeneralization, clinician trust
Pathology	Digital histopathology, cancer detection	Increased accuracy, faster turnaround	Interpretability, validation standards
Allied Health	Remote rehab, nutrition planning, speech therapy	Improved care access, individualization	Limited data, uneven access to tech
Administrative Tasks	Scheduling, billing, report generation	Operational efficiency, reduced burden	Interoperability, data privacy

#### 3.1.1 Plastic and reconstructive surgery

Artificial intelligence has demonstrated potential in preoperative planning, risk assessment, outcome prediction in surgical simulation. Nevertheless, when operating in the field of plastic surgery AI-assisted robots enable increased precision and technical capabilities especially in the field of microsurgery. AI's image processing capabilities are used to objectively assess postoperative symmetry, volume, and aesthetic outcomes. Furthermore, AI-driven diagnostic systems are aiding in the early identification of complications such as flap ischemia, often detecting issues sooner than traditional clinical methods (14).

#### 3.1.2 General surgery

The field of general surgery includes high risk operations where dexterity is vital. Artificial intelligence allows intra operative risk stratification and assistance by allowing real time identification of anatomical structures and offering surgeons guidance during various complex procedures reducing the potential error (15). A systematic review by Yagi et al. explored the role of real time instrument tracking on personalised surgical training enhancing the technical proficiency and clinical outcomes (16).

Preoperative decision-making has also been significantly improved through AI's capacity to enhance existing risk prediction models. There are several established scoring systems such as APACHE III and POSSUM and they remain fundamental tools in assessing surgical morbidity and mortality. However it has been highlighted by many conditions in the field that those systems don't take individualised risk into consideration (17, 18). Machine learning can refine these models, increasing their predictive power and allowing for more individualized risk assessment (19).

#### 3.1.3 Gastrointestinal surgery

AI has delivered some of its most impactful innovations in gastrointestinal (GI) surgery, particularly in the realm of endoscopic imaging. Endoscopic ultrasound (EUS), a key modality for differentiating between pancreatic cancer and chronic pancreatitis, has seen its diagnostic precision substantially improved through AI-based models (20–22).

In lower GI endoscopy, AI-assisted colonoscopy has shown clear clinical benefit. Computer-aided detection (CADe) systems have improved the adenoma detection rate and facilitated the differentiation of benign vs. malignant lesions (23). A randomized controlled trial further confirmed a significant increase in adenoma detection with AI-guided colonoscopy compared to conventional methods (24). Similarly, AI tools applied to upper GI endoscopy have achieved 89% accuracy (90% sensitivity and 88% specificity) in identifying neoplastic Barrett's esophagus, enhancing early detection and intervention (25).

#### 3.1.4 Oncological surgery

In oncologic surgery, artificial intelligence has been able to predict outcomes in patients with cancers by incorporating multiple tumour related and patient related variables. The models had strong prediction of patient survival and was able to identify variable that had impact on outcomes. This was achieved by integrating clinical, imaging and history pathological data enabling a tailored treatment strategy making a substantial leap forward in the practise of precision oncology (20).

#### 3.1.5 Surgical education and skill assessment

AI is redefining surgical education across disciplines. Through computer vision, AI can track surgeon hand and instrument movements in real-time, offering performance metrics and feedback that are both objective and scalable. Such technology holds the potential to standardize training globally and raise the bar for surgical competency. Machine learning and its subcategories including supervised learning, unsupervised learning, reinforced learning, and deep learning as well as various sensors including optical, inertial, electromagnetic, ultrasonic and hybrid sensors offer unique strengths and it could be applied in various surgical training and patient care settings (16).

The incorporation of AI into surgical practice offers a transformative potential across all stages of the surgical process, from preoperative risk assessment to intra-operative guidance and postoperative outcomes. As the technology matures, its thoughtful integration into clinical workflows will be crucial. Continued research, rigorous clinical trials, and strong regulatory oversight are essential to maximize its benefits while ensuring safe and ethical application which is discussed further in this review.

### 3.2 AI in medical specialties

AI technologies have been integrated into numerous medical specialties, each benefiting from improved diagnostic accuracy, predictive modeling, and personalized treatment planning. The following section classifies current applications by physiological system or clinical domain.

#### 3.2.1 Cardiovascular system

AI has been instrumental in cardiovascular diagnostics and risk prediction. Atrial fibrillation (AF) detection was among the earliest and most impactful applications. The REHEARSE-AF trial demonstrated that AF was more accurately detected using Kardia, an AI-enabled mobile ECG device, compared to routine care (26). Although wearable ECG devices have been critiqued for high false-positive rates (27), they remain valuable tools for large-scale screening. Additionally, AI applied to electronic health records (EHRs) has outperformed traditional risk calculators in predicting cardiovascular conditions such as acute coronary syndrome and heart failure (28).

#### 3.2.2 Neurological system

In neurology, AI-powered wearable sensors have proven useful in monitoring and assessing motor symptoms associated with Parkinson's disease, Huntington's disease, and multiple sclerosis (29). These devices quantify gait abnormalities, tremors, and posture with high sensitivity, supporting both diagnosis and disease progression tracking. AI also shows promise in seizure monitoring for epilepsy. Continuous ambulatory systems powered by AI algorithms can detect seizure events more reliably than conventional methods (28).

#### 3.2.3 Gastrointestinal system (endoscopy & imaging)

Artificial intelligence has significantly enhanced the diagnostic precision when it comes to diagnosing gastrointestinal pathologies. For example, the detection of colonic polyps I'm being able to distinguish whether they are benign and malignant using artificial intelligence has a higher accuracy compared to the normal clinician (23). A randomized controlled trial showed a substantial improvement in adenoma detection rates when using AI compared to standard colonoscopy (24). In upper gastrointestinal diagnostics, AI has been employed to distinguish neoplastic from non-dysplastic Barrett's esophagus with an accuracy of 89% (90% sensitivity, 88% specificity) (25).

#### 3.2.4 Oncology

Precision medicine is a clinical approach of choice, aiming to customise the treatment based on the genomic profile of the individual patient with tumour. Imagine that computational method that is able to predict the drug response based on the genomic profile of the target cells. I study by Huang et al. Revealed that artificial intelligence models were able to predict patient responses with more than 80% accuracy. The high positive predictive value suggests that a I could be used to identify promising second line treatment for options failing standard of care first line therapies (30, 31).

#### 3.2.5 Mental health

In the mental health domain, it is important to identify organised treatment programmes an monitor the treatment that he rents and guidance from mental health specialists. AI-assisted platforms support early detection, risk stratification, and treatment planning. AI-enhanced online cognitive behavioral therapy (CBT) tools have shown clinical effectiveness in treating common mental health disorders (32). Furthermore, AI models can analyze behavioral patterns and linguistic cues to assist clinicians in diagnosing depression, anxiety, and schizophrenia (9).

#### 3.2.6 Radiology and medical imaging

Medical imaging is indispensable in providing diagnostic information but it is heavily dependent on clinical interpretation and is subject to an increasing resource challenges. Automated diagnosis from medical imaging, through artificial intelligence is the future. Many studies have revealed that deep learning models have matched and exceeded human diagnostic performance leading to considerable excitement amongst clinicians and scientists. Despite the promises, a 2019 meta-analysis revealed that 99% of these studies lacked rigorous methodology, limiting their clinical reliability (33). These findings underscore the critical need for high-quality clinical trials to validate AI applications in radiology before routine adoption.

#### 3.2.7 Pathology

Artificial intelligence is being used in cancer diagnosis, providing faster, higher quality and accurate diagnosis. With the help of advanced artificial intelligence, algorithms on diagnostic techniques are being used to assist, augment and empower the computational histopathology. Whole slide imaging scanners are now providing high resolution images for the entire glass slides, combining them with digital pathology tools integrating all aspects off pathology reporting including anatomical clinical and molecular pathology (34, 35).

#### 3.2.8 AI applications in allied healthcare professions

It is indispensable to mention that artificial intelligence is increasingly being used by various allied health professionals including, physiotherapy, dieticians, speech and language therapist, and mental health providers. For instance, wearable AI devices are being utilized by physiotherapist to support remote rehabilitation and analysis of gait. Dieticians are also creating personalized nutrition plans based on individual genetics and lifestyle. Furthermore, speech and language therapists are using artificial intelligence to detect language impairment and to detect progress (36).

#### 3.2.9 Generative AI and large language models (LLMs) in clinical practice

Generative artificial intelligence and large language models, including ChatGPT, Med-PaLM and BioGPT, are increasingly being utilised in healthcare systems. They are being used to summarise clinical notes, generate discharge summaries, transcribe dictations, and even assist in decision making. A recent study published by Danqing Hu et al. demonstrated the ability of large language models to interpret radiology scans and they were found to produce impressions with high completeness and correctness but fell short in conciseness and verisimilitude, indicating that the traditional physician cannot be replaced yet (37).

### 3.3 AI in biomedical research and innovation

With limited resources available, public health and population health strategies rely on prospective analytical data to aid in guiding health initiatives effectively. This could be potentially fundamental in using predictive analytics to successfully identify patients at higher risk of developing chronic health conditions such as endocrine disorders like type 2 diabetes or cardiac conditions like heart failure. AI technology can be used to develop effective algorithms to more precisely analyse data and develop a more robust predictive model; which can reduce costs and improve patient prognoses (9). With analysis of data such as a patient's medical history and lifestyle factors, predictive models can assist in utilising targeted interventions to patients who are deemed at higher risk (38).

### 3.4 AI in biomedical research

Among the most transformative roles of artificial intelligence (AI) in healthcare is its application in biomedical research. As a driver of innovation, AI is streamlining and revolutionizing multiple stages of the research pipeline, from discovery through clinical translation.

#### 3.4.1 Drug discovery and repurposing

A landmark example is DeepMind's AlphaFold, which has significantly advanced protein structure prediction, an essential component in target identification for drug development (39). Artificial intelligence on the and the deep learning network has demonstrated ability to identify distinct antibacterial molecule structure allowing for the expansion of the antibiotics arsenal. Machine learning models are now employed to design new molecules, predict drug-target interactions, and assess toxicity and pharmacokinetics in silico, reducing the time and cost associated with early-stage drug discovery (40, 41).

#### 3.4.2 Clinical trial optimization

AI contributes to the efficiency and success of clinical trials by supporting patient recruitment, through phenotype matching and stratification, Through predictive modelling. Variable devices and mobile apps have also been used to monitor adherence. The large language models also allow for the search of unstructured clinical data increasing the inclusivity and accuracy of the trial. Predictive modeling enables adaptive trial designs, allowing dynamic modifications based on interim data, which can increase trial efficiency and reduce operational costs (42).

#### 3.4.3 Genomics and precision medicine

AI, particularly deep learning, is central to the analysis of large-scale genomic data. It facilitates the identification of pathogenic variants, enables patient stratification based on molecular signatures, and informs personalized treatment strategies. These advances are pivotal in the development of precision medicine initiatives (43, 44).

#### 3.4.4 Natural language processing in scientific literature

Natural language processing (NLP) tools allow AI systems to rapidly analyze and synthesize findings from vast repositories of scientific literature, by recognising and summarising key findings. These system can't accelerate systematic reviews and meta-analysis enabling clinicians and non-clinicians to keep up to date with the literature. This enhances hypothesis generation, accelerates systematic reviews, and supports evidence-based clinical decision-making by providing real-time insights into emerging research trends (45).

#### 3.4.5 Promoting equity in research

Recent efforts have focused on leveraging AI to reduce bias and improve inclusivity in biomedical research. AI tools are being developed to identify and correct demographic underrepresentation in clinical datasets, thereby improving the generalizability and equity of research findings across diverse populations. Machine learning models usually learn from historically collected data. Thus, data that has already experienced human biases in the past will be susceptible to incorrect predictions or withholding of resources (46). This concludes us that machine learning systems should be used proactively to advance equity. This could be achieved by incorporating distributive justice to the model design and deployment.

## 4 Limitations of AI in healthcare

AI has demonstrated its ability to enhance medical practice across many different fields. Despite this, it has been met with certain resistance, particularly from healthcare professionals more so than from patients (28).

Firstly, one needs to consider a medico-legal framework in which AI applies. From an ethical viewpoint, there needs to be a degree of accountability, particularly for errors that are made. The current regulations in place already make it a difficult task to validate clear lines of responsibilities where a medical error has occurred (7), and it becomes even less clear with AI systems. This is certainly a key area that will need close collaboration with legal authorities, healthcare staff and other key stakeholders in healthcare in order to have more clarification than there currently is.

Healthcare staff should not just be closely involved in the development of further AI services in healthcare, they should lead it. This will ensure that any data generated from algorithms can be scrutinised and therefore allow for a fairer degree of responsibility (7). Further to this, allowing clinicians to be more involved in the testing of and design of AI applications can help build a sense of trust with the system in use. Clinicians historically adopt any new technology in healthcare slowly and rely on tried and trusted methods for clinical care (47), however, with more involvement early on, this could hopefully reduce the barriers in implementing a new system for clinicians to use in everyday care.

A different general argument of the rapid introduction of AI in healthcare is the general lack of training and education in the field of digital medicine (48). There is concern about there being a general unpreparedness for this shift due to the lack of education in this field. There is also fear of AI taking the place of clinicians and “taking over”; however, more recent opinion is that AI will be complementing and contributing to clinician ability and intelligence in the future (44–50).

Other challenges include reliance on institution-specific data, which may not translate well across healthcare systems; the risk of models losing accuracy over time as practice patterns change; difficulties linking AI with existing electronic records; and the potential to add rather than reduce workload if systems are poorly integrated. These issues highlight the need for careful validation before widespread use.

### 4.1 Potential for future

There has been a clear seismic shift toward digital medicine and AI technologies incorporated in healthcare, particularly in recent times. It seems a hugely opportunistic time to guide future systems to automate and overall improve healthcare delivery.

We expect artificial intelligence to support public health and analysing patient data and environmental factors predicting potential diseases. We also expect AI to have improved ability for analysis of medical images such as x-rays MRI's and CT scans improving the sensitivity and specificity of for those tests. Other areas include personalised medicine where you receive treatment based on your genetic makeup. Artificial intelligence will also be applied in administrative tasks to streamline billing son appointment scheduling and answering patient queries (51). We will also be able to collect patient data through wearable devices and rely on telemedicine platforms for remote consultations.

There is also the consideration of the ethical implications of the wide incorporation of AI in healthcare. From a revenue perspective, it is one of the most promising markets of the modern day, with a market value reaching a thousand billion dollars in 2019 (28). A growing percentage of revenue comes from sales of medical devices, such as the ECG monitors. Governments and insurance companies are therefore striking deals with these companies. The ethical implications of medical monitoring are frequently discussed, with the potential for violation of privacy and the ongoing monitoring posing a risk to increase stigma against more disadvantaged patients or patients with more chronic illnesses (52). Data protection and ownership are clear concepts that should be strongly considered to mitigate these risks going forward.

Several different universities have created new medical curriculums to start addressing the need to educate future medical leaders about the challenges faced with AI systems (53). Both healthcare institutions and society as a whole could greatly benefit from these clinicians with a broader skillset to not only act as a safety tool for AI systems in clinical delivery but also to drive further future research in this field (28).

There is a massive potential for further cost-efficiency in healthcare with the use of AI. Although there are relatively limited cost-benefit reports currently for the use of AI in healthcare (54), there are specific examples where successful cost-effectiveness has been demonstrated.

Using AI for personalized medicine and developing predictive algorithms to forecast each patient's response to medical or surgical treatment by evaluating their genetic and environmental factors can be an effective strategy for optimizing treatment outcomes (55).

AI can play a fundamental role in drug development and manufacturing; with dose optimisation and recognition of adverse drug reactions (56) which can enhance treatment outcomes and patient safety. By utilising AI algorithms, the process of optimizing medication dosages tailored to each individual will not only improve patient safety but improve on efficiency and cost saving targets for healthcare providers. The development of new drugs and their entry into the market have been accelerated by the use of AI (57).

Furthermore, one of the greatest potentials for AI use is in robotics, with different types of robots such as mobile autonomous and educational robots being used in healthcare (7). The wider use of robotics can contribute to further cost-effectiveness. Surgical robots are becoming more in use, and as such, common minor surgical procedures may well be led by robotic systems in the future (58, 59).

Future directions include: (1) Explainable AI to improve trust; (2) Federated learning to protect privacy while training across institutions; (3) Wider integration with robotics; (4) AI tailored for low-resource settings; and (5) Green AI to reduce environmental impact. These avenues require international collaboration and robust policies.

### 4.2 Ensuring responsible AI: data integrity, privacy, and bias mitigation

The integration of AI into healthcare demands systems that are reliable, safe, and transparent, grounded in strong ethical principles and values. This requirement is a key reason why the adoption of AI in healthcare has lagged its technological advancements. As a high-stakes domain, healthcare involves sensitive and complex data, including electronic health records, medical imaging databases, wearable device outputs, and public health datasets, which necessitate careful handling and regulation (60).

Given that personal medical information is among the most private and legally protected forms of data, there are significant concerns regarding how it can be accessed, controlled, or used, particularly for training AI systems, until truly autonomous, self-training AI models are developed. A key requirement is the robust anonymisation of data, which involves removing all identifiable information, including personal details and patient record numbers.

In the European Union, such practices must comply with the General Data Protection Regulation (GDPR), which mandates transparency, informed consent, and a clear legal basis for processing this type of sensitive data (61).

Consent is not always required for AI training when using patients data under the GDPR however GDPR strictly applies if the data is not fully anonymised. According to the information Commission office it's very important to differentiate between artificial intelligence development and deployment phase this is because they are distinct and separate purposes and they involve different laws for example if an AI system was developed for a general purpose task and then you deploy it in a different context as an example a facial recognition system that could be trained to recognise recognise faces and this can be applied in many places such as prevention of crimes authentication tagging friends in social media but each application requires a different law. Processing of personal data for the purposes of training of the artificial intelligence model may not directly affect the individuals but once the model is deployed it automatically makes decisions which have significant effects and so consequences as a result law also applies here.

Under the General Data Protection Regulation (GDPR), consent is not always required for training artificial intelligence (AI) models using patient data—provided the data is fully anonymised. However, GDPR applies strictly when data is not fully anonymised, as such data is still considered personal and subject to protection.

According to the UK Information Commissioner's Office (ICO), it is crucial to distinguish between the development and deployment phases of AI. These phases represent distinct purposes and are governed by different legal considerations. For example, an AI system trained for a general task—such as facial recognition—may later be deployed in different contexts, including crime prevention, authentication, or social media tagging. Each of these applications involves separate legal frameworks and must be assessed independently for compliance.

Importantly, while the training phase of an AI model may not have a direct impact on individuals, its deployment phase often does. Once deployed, AI systems can automatically make decisions that have significant effects on individuals, such as determining eligibility for services or influencing legal outcomes. As a result, GDPR and other laws apply at the deployment stage, especially where automated decision-making or profiling is involved (62).

One critical yet underexplored aspect in the literature is algorithmic bias in artificial intelligence (AI). AI systems behave according to the data on which they are trained. If an AI is trained on datasets that reflect existing societal inequalities, it may perpetuate or even amplify those biases. For instance, an AI model trained predominantly on data from white male patients may produce inaccurate or unfair outcomes when applied to female or Black patients. Addressing this issue requires intentional diversification of training datasets, regular bias audits, and model adaptation when transferring AI systems across different populations or institutions. One approach is to fine-tune models using representative data from the new target population or allow the AI to train across multiple heterogeneous datasets. However, this raises concerns around data privacy, governance, and patient consent, especially when multiple health records are involved. Furthermore, many AI systems operate as “black boxes,” providing outputs without transparency regarding how decisions were made. This has led to growing interest in explainable AI (XAI), which aims to enhance model interpretability and ensure clinicians and patients can understand the rationale behind algorithmic decisions. XAI aims to bridges the gap between decision-making and the human interpretation of outputs (63).

The World Health Organization's guidance represents the collaborative work of global experts in ethics and digital technology, offering a framework for the responsible use of artificial intelligence in public health. It supports governments in carrying out essential public health functions, including disease surveillance, while placing ethics, human rights, and equity at the core of AI system design, deployment, and implementation (64).

The EU artificial intelligence act classifies AI systems by risk and obligates transparency, assurance of quality and traceability, especially when AI is applied in high-risk settings such as in healthcare. The medical device regulation identified the risk of the utilization of AI by further requiring conformity assessments and CE marking before utilization. In the United States, the confidentiality and breach protection, when applying AI systems, is governed by the Health Insurance Portability and Accountability. These frameworks are not there to stop the so called (progression in technology) but to ensure accountability (65–67).

## 5 Conclusion

This review evaluates the use of AI in healthcare, with emphasis on underrepresented fields. By incorporating Generative AI and allied health applications, we highlight its expanding role in an evolving healthcare landscape. As with any novel tool introduced into clinical practice, concerns remain regarding the limited initial evidence base. Despite multiple successes, resistance and lack of trust toward AI's benefits persist. Yet, its rapid adoption in other sectors demonstrates the transformative potential of this technology, and its pace of advancement has been remarkable. In the post–COVID-19 era, where healthcare faces rising demands and constrained resources, this is an opportune moment to integrate AI to strengthen service delivery. While the narrative review approach introduces the possibility of selection bias and reduced reproducibility, the multidisciplinary expertise of the authors ensured a balanced and comprehensive synthesis.
